# Association between neutrophil-to-lymphocyte ratio and cerebral small vessel disease: a systematic review and meta-analysis

**DOI:** 10.3389/fneur.2026.1748081

**Published:** 2026-07-30

**Authors:** Qian You, Ying Li, Lin Lei, Hongtao Hu

**Affiliations:** Department of Neurology, Beijing Jishuitan Hospital, Capital Medical University, Beijing, China

**Keywords:** cerebral microbleeds, cerebral small vessel disease, lacunes, neutrophil-to-lymphocyte ratio, white matter hyperintensities

## Abstract

**Background and objectives:**

Cerebral small vessel disease (CSVD) causes about 25% of ischemic strokes, most intracerebral hemorrhages, and is a major cause of vascular dementia. The neutrophil-to-lymphocyte ratio (NLR), an inflammatory marker, has shown inconsistent associations with CSVD. We performed a meta-analysis to quantify the association between NLR and CSVD imaging markers.

**Methods:**

Pubmed, Embase, and Cochrane Library were searched through August 10, 2025 for studies of NLR and CSVD. Fifteen studies (*n* = 62,961) were included. Outcomes included CSVD presence, total CSVD burden, and imaging subtypes (white matter hyperintensities [WMH], lacunes, microbleeds [CMBs], enlarged perivascular spaces [EPVS]).

**Results:**

Elevated NLR was associated with presence of CSVD (OR 1.43, 95% CI 1.07–1.93) and greater total CSVD burden (OR 1.55, 95% CI 1.11–2.18). NLR also had associations with WMH (severe WMH: OR 1.36, 95% CI 1.04–1.76; WMH volume: β=0.08, 95% CI 0.02–0.15) and lacunes (OR 1.26, 95% CI 1.16–1.36); it was not associated with CMBs (OR 0.94, 95% CI 0.82–1.09), and EPVS data were limited. Substantial heterogeneity was noted for CSVD and WMH but not for lacunes or CMBs. Removing one study resolved heterogeneity for total CSVD burden, and excluding a high-bias study eliminated the WMH severity association.

**Conclusion:**

Higher NLR showed a limited but consistent association with ischemic CSVD markers, particularly WMH and lacunes, whereas no association was observed with hemorrhagic markers such as CMBs. NLR may therefore reflect inflammatory pathways more closely related to ischemic small vessel injury, but larger longitudinal studies are needed to confirm its causal and clinical relevance.

**Systematic review registration:**

https://www.crd.york.ac.uk/PROSPERO/view/CRD420251125195, identifier: CRD420251125195.

## 1 Introduction

Cerebral small vessel disease (CSVD) accounts for approximately 25% of ischemic strokes and for the majority of spontaneous intracerebral hemorrhages. It is also the most common cause of vascular dementia and may present as vascular parkinsonism, gait disturbance, falls, and behavioral changes (1, 2). CSVD markedly impairs quality of life and functional outcomes, imposing a substantial burden on patients, families, and society.

Its prevalence increases with age, affecting about 5% of individuals over 50 years and nearly all of those over 90 years (2). Magnetic resonance imaging (MRI)-based neuroimaging is one of the principal diagnostic approaches; characteristic findings include white matter hyperintensities (WMH), small subcortical infarcts, lacunes, enlarged perivascular spaces (EPVS), and cerebral microbleeds (CMBs) (3). Importantly, these neuroimaging markers reflect different manifestations of small vessel pathology. Ischemic features, including WMH and lacunes, and hemorrhagic features, particularly CMBs, may differ in their underlying mechanisms, vascular risk factors, clinical presentations, and prognostic implications. For example, a previous study comparing lacunar infarction and subcortical hemorrhage found that ischemic and hemorrhagic consequences of CSVD showed distinct associations with vascular risk factors, neurological symptoms, in-hospital complications, and early outcomes. Given the high prevalence, substantial disease burden, and phenotypic heterogeneity of CSVD, its pathogenesis remains complex, and there are still no reliable early screening biomarkers or disease-specific interventions. Therefore, identifying accessible blood biomarkers that may help characterize CSVD and its distinct imaging phenotypes is of great significance (4).

Neuroinflammation is increasingly recognized as an important contributor to the pathogenesis, progression, and complications of CSVD. Multiple systemic inflammatory markers, including C-reactive protein (CRP), interleukin-6, and fibrinogen, have been shown to be associated with the severity of the disease (5). Potential mechanisms involve activation of microglia and astrocytes, vascular endothelial injury, dysfunction of the neurovascular unit, and increased blood-brain barrier permeability (6). These results suggest that inflammatory biomarkers may aid in the early diagnosis and risk stratification of CSVD.

The neutrophil-to-lymphocyte ratio (NLR) is a low-cost and widely available index of systemic inflammation obtained from routine blood counts. Prior studies have shown elevated NLR was associated with worse functional outcome following ischemic stroke (7, 8). In CSVD, higher NLR levels have been linked to greater disease burden (9, 10) and cognitive decline (11); however, other studies have reported no consistent associations with specific imaging phenotypes (e.g., lacunes or cerebral microbleeds) (12, 13). Overall, the evidence remains heterogeneous, and effect sizes have not yet been synthesized quantitatively. This systematic review and meta-analysis will quantify the adjusted associations between NLR and CSVD, including its neuroimaging subtypes, across diverse study designs and settings. By elucidating the links between this peripheral immune cell–derived index and CSVD severity, it may provide new evidence for the inflammatory mechanisms underlying CSVD and offer insights for therapeutic strategies and prognostic assessment.

## 2 Methods

### 2.1 Protocol and registration

The study protocol was registered prior to the data extraction process (PROSPERO registration number: CRD420251125195), and the meta-analysis was conducted in accordance with the Preferred Reporting Items for Systematic Reviews and Meta-Analyses (PRISMA) guidelines.

### 2.2 Search strategy

We systematically searched Medline, Embase, and the Cochrane Library following PRISMA guidelines covering all records published from database inception through August 10, 2025. In addition, the reference lists of eligible studies and relevant reviews were manually searched to identify further studies. No restrictions on language or publication year were applied. Search strategy in PubMed: “neutrophil-to-lymphocyte ratio”[tiab] OR “neutrophil to lymphocyte ratio”[tiab] OR “neutrophil lymphocyte ratio”[tiab] OR ((“Neutrophils”[Mesh] OR neutrophil^*^[tiab]) AND (“Lymphocytes”[Mesh] OR lymphocyt^*^[tiab]) AND ratio[tiab])) AND (“Cerebral Small Vessel Diseases”[Mesh] OR “cerebral small vessel disease”[tiab] OR “small vessel disease”[tiab] OR CSVD[tiab] OR “Leukoaraiosis”[Mesh] OR leukoaraiosis[tiab] OR “white matter hyperintensit^*^”[tiab] OR WMH[tiab] OR “white matter lesion^*^”[tiab] OR “white matter change^*^”[tiab] OR “Stroke, Lacunar”[Mesh] OR “lacunar infarct^*^”[tiab] OR “lacunar stroke^*^”[tiab] OR lacune^*^[tiab] OR “cerebral microbleed^*^”[tiab] OR microbleed^*^[tiab] OR “micro-bleed^*^”[tiab] OR “cerebral microhemorrhag^*^”[tiab] OR “cerebral microhaemorrhag^*^”[tiab] OR “perivascular space^*^”[tiab] OR EPVS[tiab] OR “Virchow Robin”[tiab] OR “Virchow-Robin”[tiab] OR “enlarged perivascular space^*^”[tiab].

The search strategies for Cochrane and Embase are provided in Supplementary material. Two reviewers (QY and YL) performed the search independently, and disagreement was resolved by consulting a third author (LL).

### 2.3 Eligibility criteria

We included studies enrolling adults (≥18 years) from either community-based or clinical populations. Eligible study designs were cohort, case-control, and cross-sectional studies that investigated the association between NLR and neuroimaging markers of CSVD, provided that a clear definition of CSVD was reported. MRI was required to assess the presence and severity of CSVD or its imaging subtypes, including WMH, lacunes, EPVS, and CMBs. Duplicate records were identified and removed manually based on title, authors and journal. When multiple publications appeared to be based on overlapping datasets, we compared study characteristics, participant characteristics, and CSVD assessment methods. If substantial overlap was suspected, we retained the study with the largest sample size; if sample sizes were identical or highly similar, the most recent report was selected. For studies reporting NLR in quantiles with the lowest quantile (Q1) as reference, we prespecified pooling of the highest vs. lowest categories as the primary analysis to avoid within-study double counting.

Only original research articles were included, while reviews, case reports, case series, animal studies, study protocols, and guidelines were excluded. Additional exclusion criteria were as follows: (1) studies explicitly recruiting participants with active infections or sepsis, hematological malignancies, solid tumors undergoing systemic therapy (chemotherapy, immunotherapy, or targeted therapy), or systemic autoimmune diseases; (2) studies of white matter lesions due to other causes such as demyelinating diseases of the central nervous system (e.g., multiple sclerosis, progressive multifocal leukoencephalopathy); (3) studies focused on genetic CSVD; (4) studies including only children or adolescents; and (5) studies in which relevant data could not be obtained.

### 2.4 Data extraction

Two reviewers (QY and YL) independently screened titles and abstracts, assessed full-text articles, and extracted data using a predesigned extraction form. Extracted information included study characteristics (first author, year of publication, study design, and sample size); population characteristics (age, sex distribution, prevalence of hypertension, diabetes, and hyperlipidemia, as well as recruitment setting); CSVD assessment methods and definitions (including WMH, lacunar infarcts, CMBs, and EPVS); outcome measures and effect estimates; and adjusted covariates. When multiple effect estimates were reported, we extracted the estimate adjusted for the greatest number of covariates. After independent extraction, the two reviewers cross-checked all extracted data, and disagreements were resolved through discussion with a third reviewer (LL).

### 2.5 Quality assessment

Two reviewers (QY and YL) independently assessed the risk of bias using the JBI Critical Appraisal Checklist for Analytical Cross-Sectional Studies (14) and the Newcastle–Ottawa Scale (NOS) for cohort and case–control studies, with disagreements resolved by a third reviewer (LL). Following JBI guidance, each item was rated as “Yes,” “No,” “Unclear,” or “Not applicable,” and results were reported item by item. For cohort and case–control studies, the NOS was applied, covering three domains (selection, comparability, and outcome), with total scores ranging from 0 to 9. A study was considered at high risk of bias if any critical JBI item (exposure validity, outcome validity, confounder identification/control, reliability, or statistical analysis) was rated “No,” or if two or more critical items were rated “No/Unclear.” For the NOS, a total score < 7 was defined as high risk of bias.

### 2.6 Statistical analysis

All analyses were performed using Stata version 14.0 (StataCorp, College Station, TX, USA). Adjusted effect estimates were meta-analyzed with random-effects models. Heterogeneity was assessed using the I^2^ statistic, with substantial heterogeneity defined as I^2^ >50%. Sensitivity analyses were conducted by excluding studies judged to be at high risk of bias. When fewer than three studies were available for a given outcome, results were narratively synthesized without pooling. Potential publication bias was assessed using funnel plots and Egger's or Harbord's tests when ≥10 studies were available.

## 3 Results

### 3.1 Review of literature

**Figure 1:**
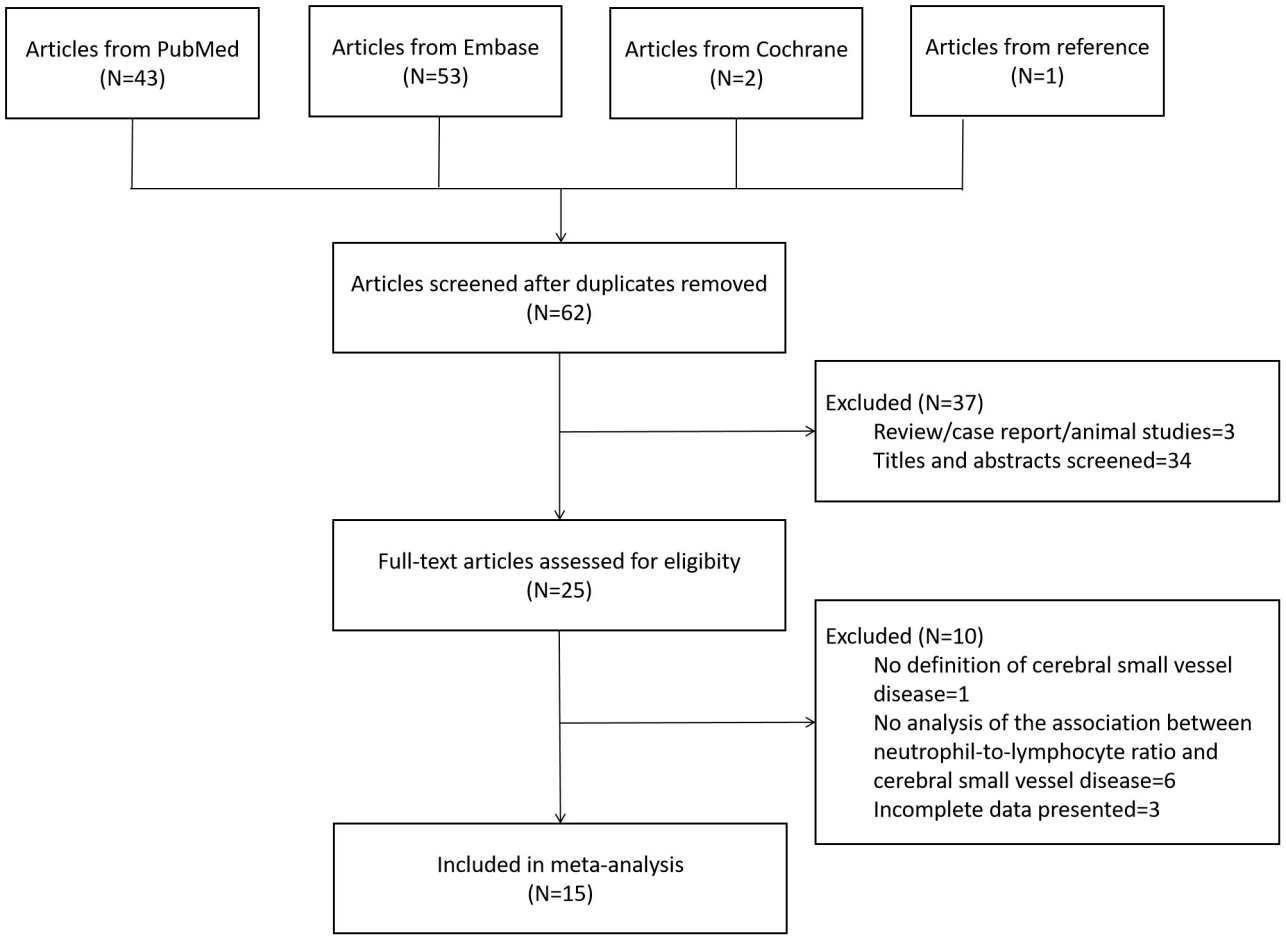
Flow diagram of study selection details. A total of 99 articles were screened for eligibility and 15 studies were included finally in the analysis.

A total of 99 records were identified through the search strategy, including 43 from PubMed, 53 from Embase, 2 from the Cochrane Library, and 1 from manual reference checking. After removal of 37 duplicates, 62 records remained. Of these, 3 were excluded as reviews or case reports, and 34 were excluded after screening titles and abstracts for irrelevance. The full texts of the remaining 25 articles were reviewed in detail. Among them, 1 study did not provide a clear definition of CSVD, 6 did not investigate the association between NLR and CSVD, and 3 lacked usable data. Ultimately, 15 studies were included in the analysis (Figure 1).

### 3.2 Study characteristics

A total of 15 studies comprising 62,961 participants were included. The median sample size was 950, ranging from 148 to 36,411. Six studies were prospective, seven were retrospective, and two did not clearly report whether they were prospective or retrospective. Nine studies were conducted in clinical settings, including three enrolling patients with acute stroke, while the remaining six were community-based. For 513 participants, age was reported only as proportions above or below 60 years; in the remaining 62,448 participants, the mean age was 56.6 ± 9.5 years. Among 62,241 participants, 31,071 were male (49.9%). With respect to vascular risk factors, 21.8% had hypertension, 6.1% had diabetes, 17.9% had hyperlipidemia, and 9.6% were smokers (Supplementary Table 1).

Regarding outcomes, four studies evaluated the association between NLR and the presence of CSVD, and four assessed its relationship with overall CSVD burden. For imaging subtypes, five studies analyzed WMH severity as a binary variable and five examined WMH volume as a continuous measure. Associations between NLR and lacunes or cerebral microbleeds were investigated in six and four studies, respectively, while only one study assessed the presence of EPVS and two studies examined EPVS severity.

### 3.3 Quality assessment

All 15 included studies were cross-sectional analyses and were therefore assessed using the JBI Critical Appraisal Checklist for Analytical Cross-Sectional Studies. Six studies were rated as “unclear” for inclusion criteria, two as “unclear” for control of confounders, and three as “unclear” for reliability. For other domains, including participants, exposure, outcome, identification of confounders, and statistical analysis, all studies were judged to be of acceptable quality. Ultimately, only one study was classified as having a high risk of bias (Supplementary Table 2).

### 3.4 Synthesis of results

#### 3.4.1 Association of NLR with the presence and burden of CSVD

**Figure 2:**
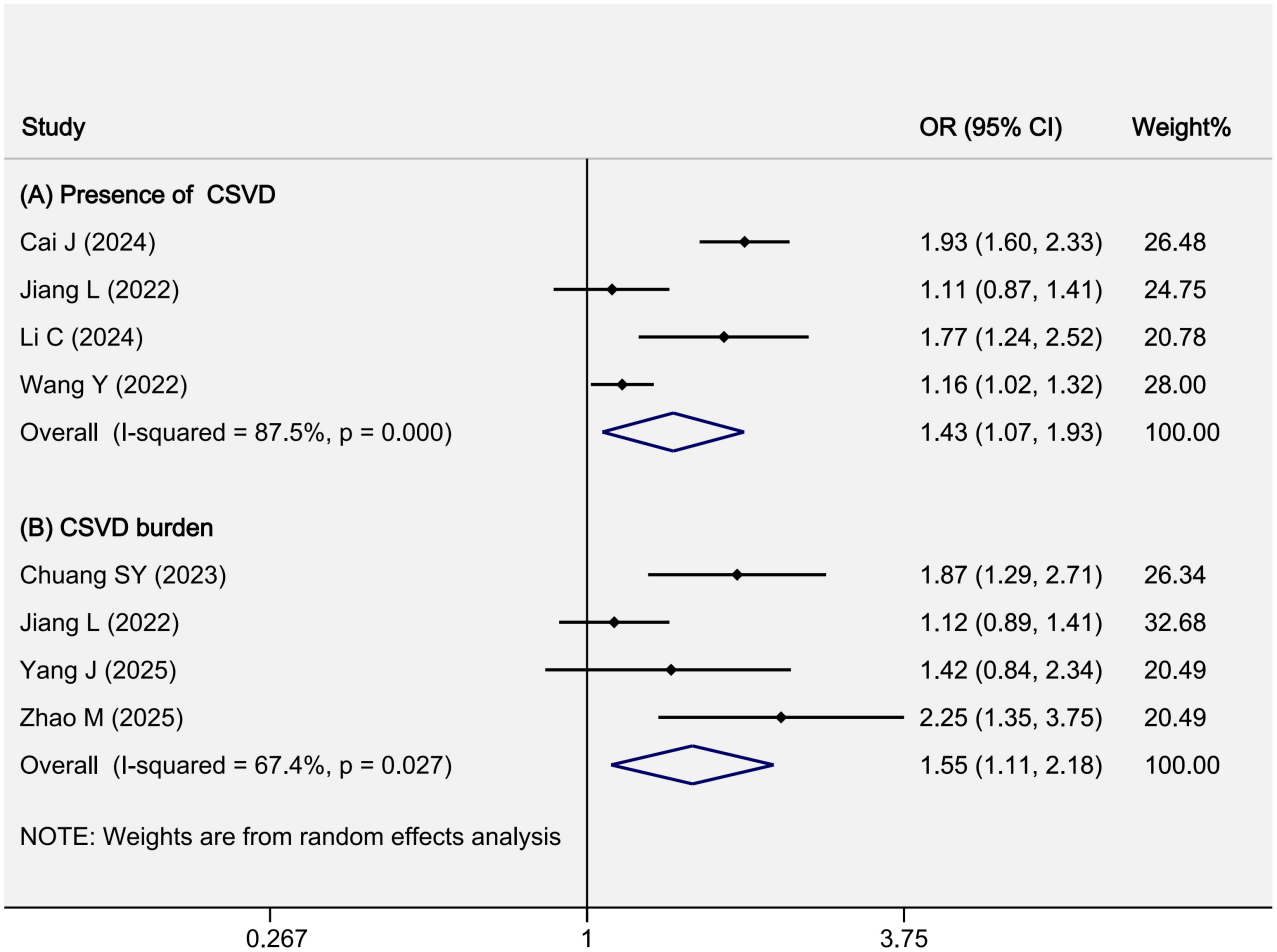
Forest plots of the association between NLR and CSVD. **(A)** Association between NLR and the presence of CSVD. **(B)** Association between NLR and the ordinal increment of CSVD burden. NLR, neutrophil-to-lymphocyte ratio; CSVD, cerebral small vessel disease; OR, odds ratio; CI, confidence interval.

Four studies including 5,445 participants evaluated the association between NLR and the presence of CSVD (10, 15–17). Three of these studies reported significant associations (OR 1.929, 95% CI 1.599–2.327; OR 1.77, 95% CI 1.24–2.52; OR 1.158, 95% CI 1.016–1.321). The pooled analysis demonstrated that higher NLR levels were associated with the presence of CSVD (OR 1.43, 95% CI 1.07–1.93), with substantial heterogeneity (I^2^ = 87.5%) (Figure 2). Sensitivity analyses showed that heterogeneity remained high regardless of which study was excluded (I^2^ ranging from 62.2% to 90.7%) (Supplementary Table 3).

Four studies comprising 12,401 participants assessed CSVD burden using total burden scores (9, 10, 18, 19). Two studies reported significant positive associations (OR 1.87, 95% CI 1.29–2.71; OR 2.246, 95% CI 1.346–3.75), while the other two found null associations. The pooled analysis indicated that higher NLR levels were associated with greater CSVD burden (OR 1.55, 95% CI 1.11–2.18), with moderate-to-high heterogeneity (I^2^ = 67.4%) (Figure 2). Sensitivity analysis revealed that exclusion of the study by Jiang et al. (10) reduced heterogeneity to 0.0%, while the association remained significant (OR 1.83, 95% CI 1.41–2.37) (Supplementary Table 3). These sensitivity analyses suggested that the association between NLR and CSVD presence should be interpreted cautiously because of persistent heterogeneity, whereas the association with total CSVD burden appeared more robust after exclusion of the main source of heterogeneity.

#### 3.4.2 Association of NLR with different neuroimaging markers of CSVD

##### 3.4.2.1 WMH

**Figure 3:**
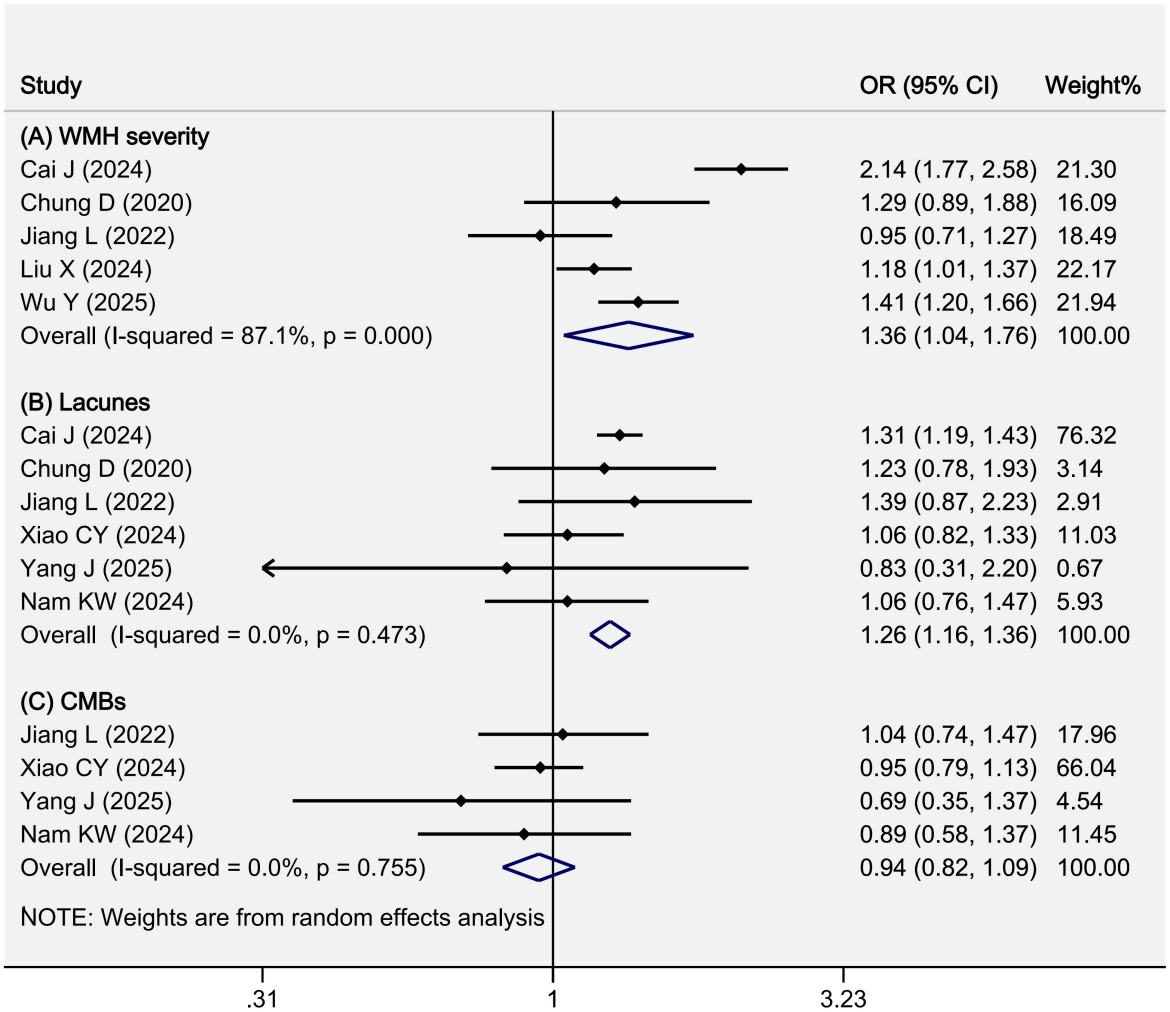
Forest plots of the association between NLR and different MRI markers of CSVD. **(A)** Association between NLR and WMH severity. **(B)** Association between NLR and the presence of lacunes. **(C)** Association between NLR and the presence of CMBs. NLR, neutrophil-to-lymphocyte ratio; CSVD, cerebral small vessel disease; WMH, white matter hyperintensities; MRI, magnetic resonance imaging; CMBs, cerebral microbleeds; OR, odds ratio; CI, confidence interval.

Five studies including 5,560 participants examined the association between NLR levels and WMH severity, dichotomized as mild vs. severe (10, 15, 20–22). Three studies reported significant associations (OR 2.136, 95% CI 1.768–2.58; OR 1.18, 95% CI 1.014–1.374; OR 1.41, 95% CI 1.20–1.66), while two reported null associations. The pooled analysis demonstrated that higher NLR levels were associated with greater WMH severity (OR 1.36, 95% CI 1.04–1.76), with considerable heterogeneity (I^2^ = 87.1%) (Figure 3). Sensitivity analyses indicated that heterogeneity remained high regardless of which study was excluded (I^2^ ranging from 50.7% to 90.3%) (Supplementary Table 4). Notably, one study was judged to be at high risk of bias; when this study was excluded, the association weakened and was no longer statistically significant (OR 1.34, 95% CI 0.92–1.93).

**Figure 4:**
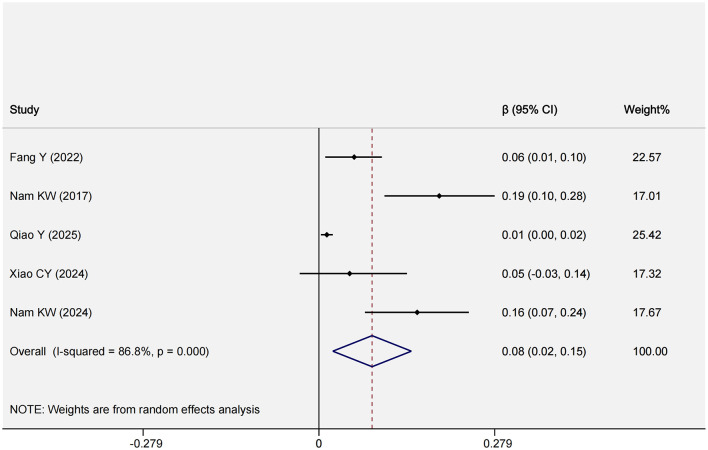
Forest plot of the association between NLR and WMH volume. Forest plot showing pooled β coefficients and 95% CIs from linear regression models evaluating the association between NLR and WMH volume measured by MRI. NLR, neutrophil-to-lymphocyte ratio; WMH, white matter hyperintensities; MRI, magnetic resonance imaging; CI, confidence interval.

Five studies involving 46,326 participants quantified WMH volume using MRI and analyzed its association with NLR levels through linear regression (13, 23–26). Four studies reported significant positive associations. The pooled analysis showed that higher NLR levels were significantly associated with greater WMH volume (β = 0.08, 95% CI 0.02–0.15), although substantial heterogeneity was observed (I^2^ = 86.6%, *p* < 0.001) (Figure 4). Sensitivity analyses demonstrated that exclusion of any single study did not alter the overall result, supporting a linear association between NLR and WMH volume; however, heterogeneity remained consistently high (I^2^ ranging from 71.7% to 89.8%) (Supplementary Table 4). Thus, sensitivity analyses indicated that the association between NLR and dichotomized WMH severity was less robust and sensitive to study quality, whereas the association with quantitative WMH volume remained relatively stable despite persistent heterogeneity.

##### 3.4.2.2 Lacunes

Six studies involving 17,477 participants evaluated the association between NLR and lacunes (10, 13, 15, 19, 20, 23). Only one study reported a significant positive association (OR 1.309, 95% CI 1.194–1.435), while the other five found no significant relationship. The pooled analysis demonstrated that higher NLR levels were associated with the presence of lacunes (OR 1.26, 95% CI 1.16–1.36), with no evidence of heterogeneity (I^2^ = 0.0%) (Figure 3).

##### 3.4.2.3 CMBs

Four studies including 15,860 participants investigated the association between NLR and CMBs, and all reported null results (10, 13, 19, 23). Accordingly, the pooled analysis also showed no significant association between NLR and microbleeds (OR 0.94, 95% CI 0.82–1.09), with no heterogeneity across studies (I^2^ = 0.0%) (Figure 3).

##### 3.4.2.4 EPVS

One study (*n* = 8,481) reported no significant association between NLR and the presence of EPVS (OR 0.92, 95% CI 0.72–1.18) (19). Two studies (*n* = 667 and *n* = 8,481) assessed EPVS severity and yielded inconsistent findings (10, 15), with one showing a positive association (OR 1.248, 95% CI 1.11–1.402) and the other reporting no significant relationship (OR 1.02, 95% CI 0.54–1.95). In addition, two studies comprising 3,719 participants found that basal ganglia EPVS were positively associated with NLR (OR 1.136, 95% CI 1.012–1.275; OR 1.68, 95% CI 1.16–2.45) (10, 15), while one study (*n* = 667) identified a significant association between NLR and centrum semi-ovale EPVS (OR 1.14, 95% CI 1.027–1.266) (15). Given the limited number of studies and the inconsistency of results, further research is needed to clarify the relationship between NLR and EPVS.

## 4 Discussion

In this systematic review and meta-analysis, we found that higher NLR levels were significantly associated with overall CSVD burden and, more specifically, with WMH and lacunes. The association was most consistent for WMH volume, whereas results for WMH severity showed considerable heterogeneity and were sensitive to study quality. A significant association was also observed between NLR and lacunes, with low heterogeneity. In contrast, no evidence supported a relationship between NLR and CMBs, and findings for EPVS were limited and inconsistent.

This study found that NLR was associated with the presence of CSVD. However, the heterogeneity was substantial, which may be attributable to variability in study design, populations, or adjustment strategies. In analyses of NLR and overall CSVD burden, heterogeneity disappeared after excluding one study (10), and the association remained robust, suggesting that the observed inconsistency was driven by methodological differences in a single study rather than by the absence of a true association. This study may have contributed to the heterogeneity because it enrolled a community-based population rather than stroke patients, who were generally healthier, had milder CSVD, and lower levels of systemic inflammation. After comprehensive covariate adjustment, the association between NLR and total CSVD burden may have been attenuated, resulting in a statistically null effect. In addition, its large sample size (*n* = 3052) gave it considerable weight in the meta-analysis, making it a major source of heterogeneity.

In the analysis of NLR and CSVD subtypes, we first examined WMH. WMH are clinically important imaging markers of CSVD, as white matter abnormalities have been associated with cognitive impairment and adverse clinical outcomes. For example, in patients with metabolic syndrome, microstructural white matter alterations assessed by diffusion tensor imaging were correlated with cognitive impairment, particularly deficits in processing speed (27). Metabolic syndrome represents a cluster of vascular risk factors, such as hypertension, dyslipidemia, glucose dysregulation, and obesity, which may affect cerebrovascular integrity during aging. These findings highlight the importance of interpreting WMH as a clinically meaningful CSVD phenotype. In our meta-analysis, higher NLR levels were significantly associated with WMH severity when categorized as mild vs. severe. However, this association disappeared in sensitivity analyses after excluding one study judged to be at high risk of bias (22). By contrast, NLR was significantly associated with quantitative WMH volume, and the results remained stable across sensitivity analyses. Taken together, these findings suggested that NLR was likely to be associated with WMH. Moreover, NLR was also associated with lacunes. As WMH and lacunes are recognized markers of chronic small vessel damage and cumulative ischemic burden, these results indicate that ischemic CSVD phenotypes may be more closely linked to systemic inflammatory pathways. In contrast, no significant association was found between NLR and CMBs, suggesting that NLR may not be related to hemorrhagic manifestations of CSVD.

Potential pathophysiological mechanisms of the association between elevated NLR and ischemic CSVD lesions were as follows. Increased NLR is a marker of systemic inflammation, reflecting increased neutrophil count and decreased lymphocyte count. Studies have shown that in patients with arteriosclerotic CSVD, circulating leukocytes, largely neutrophils, overexpress chemokine receptors, adhesion molecules and matrix metalloproteins associated with endothelial injury and blood brain barrier leakage (10, 12). Additionally, activated neutrophils elevate oxidative stress by releasing myeloperoxidase (MPO), which generates reactive oxygen species and subsequently leads to endothelial injury and impaired vasodilation (28). It has been shown that increased MPO levels were associated with lacunar stroke (28). Lymphocytes, on the other hand, produce anti-inflammatory cytokines (interleukin-4, interleukin-10, interleukin-13) and support angiogenic and reparative processes in the brain (15). This immune dysregulation, excessive neutrophil-mediated inflammation coupled with diminished lymphocyte-mediated regulation, provides a plausible link between high NLR and ongoing ischemic small vessel damage.

In contrast to the link with ischemic CSVD features, NLR has not shown a significant association with CMBs in the current study. Hemorrhagic CSVD lesions often arise from structural degenerative changes in the vessel wall rather than inflammatory processes. In cerebral amyloid angiopathy, β-amyloid deposits in the vessel walls lead to vessel fragility and microscopic hemorrhages. This is considered as a chronic degenerative process with relatively little contribution from systemic neutrophil-driven inflammation (29). Likewise, hypertensive arteriopathy causes fibrinoid necrosis in perforating arterioles, predisposing to microaneurysm rupture and microbleeds, which is a mechanism more related to prolonged hemodynamic stress than to circulating leukocyte activity. Therefore, a high NLR might not directly exacerbate the pathology that produces microbleeds.

Our study has several strengths. It was conducted according to a pre-defined protocol and was prospectively registered in PROSPERO. The review was rigorous and up-to-dated, and it systematically analyzed the association between NLR and CSVD from both an overall burden perspective and across specific neuroimaging subtypes.

This study also has several limitations. First, all included studies were cross-sectional analyses, which limited our ability to determine the temporal or causal relationship between NLR and CSVD. Second, because fewer than ten studies were included in each analysis, we were unable to formally assess publication bias. Although both positive and null studies were identified, the possibility of publication bias cannot be excluded. Third, substantial heterogeneity was observed in most pooled analyses, while the number of studies within each analysis was relatively small. This limited our ability to conduct meaningful subgroup analyses, as dividing studies into subgroups would further reduce the sample size and statistical power. Therefore, larger, well-designed studies are still needed to further clarify the relationship between NLR and CSVD. Fourth, although lacunes were analyzed as an imaging marker of CSVD, the included studies did not consistently report the clinical manifestations of lacunar syndromes. Thus, we were unable to evaluate the frequency of different clinical lacunar syndromes. Future studies should integrate detailed clinical phenotyping with MRI-defined lacunar lesions to clarify whether NLR are associated with specific lacunar clinical presentations.

Taken together, our findings support a potential role of systemic inflammation, as reflected by NLR, in the pathogenesis and progression of ischemic CSVD. Given its accessibility and low cost, NLR may serve as a pragmatic biomarker to aid in risk stratification of individuals at higher risk of CSVD-related ischemic damage. However, NLR should not be interpreted as a stand-alone diagnostic biomarker. Instead, it may be more useful as part of a multi-biomarker panel integrating other inflammatory markers, such as CRP, interleukin-6, and fibrinogen, together with conventional vascular risk factors and MRI markers. Such integrated models may improve CSVD risk stratification, but their performance and clinical utility require validation in future longitudinal studies. In addition to evaluating CSVD imaging progression, future studies should incorporate cognitive and functional outcomes to determine the clinical significance of NLR-related CSVD burden. Mechanistic studies are also needed to explore how peripheral immune dysregulation may contribute to distinct CSVD phenotypes. Moreover, sleep-related breathing disorders may be considered in future research on the relationship among NLR, systemic inflammation, and CSVD, as they have been linked to inflammatory biomarkers and stroke-related outcomes (30).

## 5 Conclusion

Higher NLR showed a limited but consistent association with ischemic CSVD markers, particularly WMH and lacunes, whereas no association was observed with hemorrhagic markers such as CMBs. NLR may therefore reflect inflammatory pathways more closely related to ischemic small vessel injury, but larger longitudinal studies are needed to confirm its causal and clinical relevance.

## Data Availability

The original contributions presented in the study are included in the article/Supplementary material, further inquiries can be directed to the corresponding author.

## References

[1] Markus HS de Leeuw FE. Cerebral small vessel disease: recent advances and future directions. Int J Stroke. (2023) 18:4–14. doi: 10.1177/17474930221144911 36575578 PMC9806465

[2] Dupre N Drieu A Joutel A. Pathophysiology of cerebral small vessel disease: a journey through recent discoveries. J Clin Invest. (2024) 134:e172841. doi: 10.1172/JCI172841 38747292 PMC11093606

[3] Wardlaw JM Smith EE Biessels GJ Cordonnier C Fazekas F Frayne R . Neuroimaging standards for research into small vessel disease and its contribution to ageing and neurodegeneration. Lancet Neurol. (2013) 12:822–38. doi: 10.1016/S1474-4422(13)70124-8 23867200 PMC3714437

[4] Bernal M Escarcena P Arboix A Garcia-Eroles L Vergés E Díez L . Differential characteristics of ischemic and hemorrhagic stroke in patients with cerebral small vessel disease. Neurol India. (2021) 69:85–90. doi: 10.4103/0028-3886.310106 33642276

[5] Low A Mak E Rowe JB Markus HS O'Brien JT. Inflammation and cerebral small vessel disease: a systematic review. Ageing Res Rev. (2019) 53:100916. doi: 10.1016/j.arr.2019.100916 31181331

[6] Wu LY Chai YL Cheah IK Chia RSL Hilal S Arumugam TV . Blood-based biomarkers of cerebral small vessel disease. Ageing Res Rev. (2024) 95:102247. doi: 10.1016/j.arr.2024.102247 38417710

[7] Gong P Liu Y Gong Y Chen G Zhang X Wang S . The association of neutrophil to lymphocyte ratio, platelet to lymphocyte ratio, and lymphocyte to monocyte ratio with post-thrombolysis early neurological outcomes in patients with acute ischemic stroke. J Neuroinflammation. (2021) 18:51. doi: 10.1186/s12974-021-02090-6 33610168 PMC7896410

[8] Li W Hou M Ding Z Liu X Shao Y Li X. Prognostic value of neutrophil-to-lymphocyte ratio in stroke: a systematic review and meta-analysis. Front Neurol. (2021) 12:686983. doi: 10.3389/fneur.2021.686983 34630275 PMC8497704

[9] Chuang SY Hsu YC Chou KW Chang KS Wong CH Hsu YH . Neutrophil-lymphocyte ratio as a predictor of cerebral small vessel disease in a geriatric community: the I-Lan longitudinal aging study. Brain Sci. (2023) 13:1087. doi: 10.3390/brainsci13071087 37509017 PMC10377025

[10] Jiang L Cai X Yao D Jing J Mei L Yang Y . Association of inflammatory markers with cerebral small vessel disease in community-based population. J Neuroinflammation. (2022) 19:106. doi: 10.1186/s12974-022-02468-0 35513834 PMC9072153

[11] Hou L Zhang S Qi D Jia T Wang H Zhang W . Correlation between neutrophil/lymphocyte ratio and cognitive impairment in cerebral small vessel disease patients: a retrospective study. Front Neurol. (2022) 13:925218. doi: 10.3389/fneur.2022.925218 35989913 PMC9391025

[12] Van der Taelen L Briones AM Unger T Staals J van Oostenbrugge RJ Foulquier S. Circulating immune cells in cerebral small vessel disease: a systematic review. Biogerontology. (2025) 26:101. doi: 10.1007/s10522-025-10250-x 40323444 PMC12052918

[13] Nam KW Kwon HM Jeong HY Park JH Min K. Monocyte to high-density lipoprotein cholesterol ratio is associated with cerebral small vessel diseases. BMC Neurol. (2024) 24:18. doi: 10.1186/s12883-023-03524-9 38178033 PMC10765827

[14] Barker TH Hasanoff S Aromataris E Stone JC Leonardi-Bee J Sears K . The revised JBI critical appraisal tool for the assessment of risk of bias for analytical cross-sectional studies. JBI Evid Synth. (2026) 24:401–8. doi: 10.11124/JBIES-24-00523 40521701

[15] Cai J Zeng X Huang X Dong H Liu J Lin J . Relationship of neutrophil/lymphocyte ratio with cerebral small vessel disease and its common imaging markers. Immun Inflamm Dis. (2024) 12:e1228. doi: 10.1002/iid3.1228 38578037 PMC10996379

[16] Li C Wang J Han X Li Y Liu K Zhao M . Development and validation of a diagnostic model for cerebral small vessel disease among rural older adults in China. Front Neurol. (2024) 15:1388653. doi: 10.3389/fneur.2024.1388653 39036632 PMC11258008

[17] Wang Y Ma L Zhang M Wei J Li X Pan X . Blood neutrophil-to-lymphocyte ratio as a predictor of cerebral small-vessel disease. Med Sci Monit. (2022) 28:e935516. doi: 10.12659/MSM.935516 35470355 PMC9057675

[18] Zhao M Xie X Hao Z Ren M Teng Z Xu J . The correlation between neutrophil/lymphocyte ratio and the MRI burden and cognitive function in patients with cerebral small vessel disease. Front Neurol. (2025) 16:1546076. doi: 10.3389/fneur.2025.1546076 40401029 PMC12092228

[19] Yang J Liang Z Li D Wang J Mei L Hu L . Analysis of the correlation between peripheral blood inflammatory markers and imaging burden of cerebral small vessel disease. Front Neurol. (2025) 16:1538028. doi: 10.3389/fneur.2025.1538028 40726623 PMC12301902

[20] Chung D Lee KO Choi JW Kim NK Kim OJ Kim SH . Blood neutrophil/lymphocyte ratio is associated with cerebral large-artery atherosclerosis but not with cerebral small-vessel disease. Front Neurol. (2020) 11:1022. doi: 10.3389/fneur.2020.01022 33013672 PMC7509145

[21] Liu X Wu N Li J Pang M Wang Y Wang Y . Risk factors and clinical characteristics of first-ever ischemic stroke caused by ICAS with leukoaraiosis. Int J Med Sci. (2024) 21:1500–10. doi: 10.7150/ijms.95984 38903919 PMC11186426

[22] Wu Y Hu J Zhao Y Ju D Cao S Guo J . The neutrophil-to-lymphocyte ratio is associated with functional outcome among single small subcortical infarction: Mediating effects of white matter hyperintensities. J Stroke Cerebrovasc Dis. (2025) 34:108341. doi: 10.1016/j.jstrokecerebrovasdis.2025.108341 40345411

[23] Xiao CY Ma YH Zhao YL Liu JY Tan L Alzheimer's Disease Neuroimaging I. Association of peripheral immunity and cerebral small vessel disease in older adults without dementia: a longitudinal study. Neurobiol Aging. (2024) 137:55–61. doi: 10.1016/j.neurobiolaging.2024.02.007 38422799

[24] Fang Y Doyle MF Alosco ML Mez J Satizabal CL Qiu WQ . Cross-sectional association between blood cell phenotypes, cognitive function, and brain imaging measures in the community-based Framingham heart study. J Alzheimers Dis. (2022) 87:1291–305. doi: 10.3233/JAD-215533 35431244 PMC9969805

[25] Nam KW Kwon HM Jeong HY Park JH Kim SH Jeong SM . High neutrophil to lymphocyte ratio is associated with white matter hyperintensity in a healthy population. J Neurol Sci. (2017) 380:128–31. doi: 10.1016/j.jns.2017.07.024 28870552

[26] Qiao Y Zhao L Cong C Li Y Tian S Zhu X . Association of systemic inflammatory markers with white matter hyperintensities and microstructural injury: an analysis of UK Biobank data. J Psychiatry Neurosci. (2025) 50:E45–56. doi: 10.1503/jpn.240039 39848683 PMC11771994

[27] Segura B Jurado MA Freixenet N Bargallo N Junque C Arboix A. White matter fractional anisotropy is related to processing speed in metabolic syndrome patients: a case-control study. BMC Neurol. (2010) 10:64. doi: 10.1186/1471-2377-10-64 20663196 PMC2920865

[28] Karel MFA Roosen MGCH Tullemans BME Zhang CE Staals J Cosemans JMEM . Characterization of cerebral small vessel disease by neutrophil and platelet activation markers using artificial intelligence. J Neuroimmunol. (2022) 367:577863. doi: 10.1016/j.jneuroim.2022.577863 35436744

[29] Liu X Sun P Yang J Fan Y. Biomarkers involved in the pathogenesis of cerebral small-vessel disease. Front Neurol. (2022) 13:969185. doi: 10.3389/fneur.2022.969185 36119691 PMC9475115

[30] Uscamaita K Parra Ordaz O Yazbeck Morell I Pla MG Sanchez-Lopez MJ Arboix A. From molecular to clinical implications of sleep-related breathing disorders on the treatment and recovery of acute stroke: a scoping review. Curr Issues Mol Biol. (2025) 47:138. doi: 10.3390/cimb47030138 40136392 PMC11941448

