# Supplementary material for: Association between neutrophil-to-lymphocyte ratio and cerebral small vessel disease: a systematic review and meta-analysis
Source: Front Neurol. 2026 Jul 30;17:1748081. doi: 10.3389/fneur.2026.1748081 (PMC13467753; doi:10.3389/fneur.2026.1748081)
Supplement: Supplementary file 1 [file Table_1.docx]

**Embase search strategy**

('neutrophil-to-lymphocyte ratio':ti,ab,kw OR 'neutrophil to lymphocyte ratio':ti,ab,kw OR 'neutrophil lymphocyte ratio':ti,ab,kw OR (('neutrophil count'/exp OR neutrophil*:ti,ab,kw) AND ('lymphocyte count'/exp OR lymphocyt*:ti,ab,kw) AND ratio:ti,ab,kw)) AND ('cerebral small vessel disease':ti,ab,kw OR 'small vessel disease':ti,ab,kw OR csvd:ti,ab,kw OR 'leukoaraiosis'/exp OR leukoaraiosis:ti,ab,kw OR 'white matter hyperintensit*':ti,ab,kw OR wmh:ti,ab,kw OR 'white matter lesion*':ti,ab,kw OR 'white matter change*':ti,ab,kw OR 'lacunar stroke'/exp OR 'lacunar infarct*':ti,ab,kw OR 'lacunar stroke*':ti,ab,kw OR lacune*:ti,ab,kw OR 'cerebral microbleed*':ti,ab,kw OR microbleed*:ti,ab,kw OR 'micro-bleed*':ti,ab,kw OR 'cerebral microhemorrhag*':ti,ab,kw OR 'perivascular space*':ti,ab,kw OR epvs:ti,ab,kw OR 'virchow robin':ti,ab,kw OR 'virchow-robin':ti,ab,kw OR 'enlarged perivascular space*':ti,ab,kw)

**The Cochrane Library search strategy**

#1	(neutrophil-to-lymphocyte ratio):ti,ab,kw OR (neutrophil to lymphocyte ratio):ti,ab,kw OR (neutrophil lymphocyte ratio):ti,ab,kw

#2	(neutrophil*):ti,ab,kw

#3	(lymphocyt*):ti,ab,kw

#4	(ratio):ti,ab,kw

#5	MeSH descriptor: [Neutrophils] explode all trees

#6	MeSH descriptor: [Lymphocytes] explode all trees

#7	#2 OR #5

#8	#3 OR #6

#9	#7 AND #8 AND #4

#10	#1 OR #9

#11	MeSH descriptor: [Cerebral Small Vessel Diseases] explode all trees

#12	(cerebral small vessel disease):ti,ab,kw OR (small vessel disease):ti,ab,kw OR (CSVD):ti,ab,kw

#13	MeSH descriptor: [Leukoaraiosis] explode all trees

#14	(leukoaraiosis):ti,ab,kw OR (white matter hyperintensit*):ti,ab,kw OR (white matter lesion*):ti,ab,kw OR (white matter change*):ti,ab,kw

#15	MeSH descriptor: [Stroke, Lacunar] explode all trees

#16	(lacunar infarct*):ti,ab,kw OR (lacunar stroke*):ti,ab,kw OR (lacune*):ti,ab,kw

#17	(cerebral microbleed*):ti,ab,kw OR (microbleed*):ti,ab,kw OR (micro-bleed*):ti,ab,kw OR (cerebral microhemorrhag*):ti,ab,kw OR (cerebral microhemorrhag*):ti,ab,kw

#18	(perivascular space*):ti,ab,kw OR (Virchow Robin):ti,ab,kw OR (Virchow-Robin):ti,ab,kw OR (enlarged perivascular space*):ti,ab,kw

#19	#11 OR #12 OR #13 OR #14 OR #15 OR #16 OR #17 OR #18

#20	#10 AND #19

**Supplemental Table 1. Baseline characteristics of the studies included in the systematic review and meta-analysis**

| Study | Study design | Clinical scene | Sample size | Age  (years) | Male  n(%) | Hypertension  n(%) | Diabetes  n(%) | Dyslipidemia  n(%) | Smoking  n(%) |
| --- | --- | --- | --- | --- | --- | --- | --- | --- | --- |
| Cai J,2024 | Retrospective | Clinic | 667 | 62±13 | 379(56.8) | 318(47.7) | 96(14.4) | / | 208(31.2) |
| Chuang SY,2023 | Prospective | Community | 720 | 63±9 | / | / | / | / | / |
| Chung D,2020 | Retrospective | Clinic | 950 | 66±9 | 347(36.5) | 540(56.8) | 211(22.2) | 298(31.4) | 201(21.2) |
| Fang Y,2022 | Prospective | Community | 2713 | 59±14 | 1286(47.4) | 916(33.8) | 254(9.4) | 882(32.5) | 196(7.2) |
| Jiang L,2022 | Prospective | Community | 3052 | 61±7 | 1419(46.5) | 1314(43.1) | 659(21.6) | 612(20.1) | 623(20.4) |
| Li C,2024 | Undetermined | Community | 847 | 69±5 | 352(41.6) | / | / | / | 169(20.0) |
| Liu X,2024 | Retrospective | Clinic | 378 | 63±11 | 241(63.8) | 205(54.2) | 100(26.5) | 40(10.6) | 125(33.1) |
| Nam KW,2017 | Retrospective | Clinic | 2875 | 56±9 | 1565(54.4) | 641(22.3) | 399(13.9) | 727(25.3) | 447(15.5) |
| Qiao Y,2025 | Prospective | Community | 36411 | 55±8 | 17496(48.1) | 7060(19.4) | 894(2.5) | / | 2272(6.2) |
| Wang Y,2022 | Retrospective | Clinic | 879 | 63±12 | 451(51.3) | 544(61.9) | 219(24.9) | 42(4.8) | 215(24.5) |
| Wu Y,2025 | Prospective | Clinic | 513 | / | 332(64.7) | 350(68.2) | 144(28.1) | 37(7.2) | 150(29.2) |
| Xiao CY,2024 | Prospective | Community | 1183 | 74±7 | 514(43.4) | 509(43.0) | 110(9.3) | 536(45.3) | 365(30.9) |
| Yang J,2025 | Undetermined | Clinic | 8481 | 55±11 | 4893(57.7) | 99(1.2) | 124(1.5) | 663(7.8) | 435(5.1) |
| Zhao M,2025 | Retrospective | Clinic | 148 | 67±9 | 100(67.6) | 102(68.9) | 47(31.8) | / | 33(22.3) |
| Nam KW,2024 | Retrospective | Clinic | 3144 | 56±10 | 1696(53.9) | 791(25.2) | 462(14.7) | 480(15.3) | 565(18.0) |

**Supplemental Table 2. Quality assessment performed using the JBI Critical Appraisal Checklist for Analytical Cross-Sectional Studies.**

| Study | Inclusion criteria | Participants | Exposure | Outcome | Confounders identified | Confounders control | Reliability | Statistical analysis | High risk |
| --- | --- | --- | --- | --- | --- | --- | --- | --- | --- |
| Cai J, 2024 | Yes | Yes | Yes | Yes | Yes | Yes | Yes | Yes | No |
| Chuang SY, 2023 | Unclear | Yes | Yes | Yes | Yes | Yes | Yes | Yes | No |
| Chung D, 2020 | Unclear | Yes | Yes | Yes | Yes | Yes | Yes | Yes | No |
| Fang Y, 2022 | Yes | Yes | Yes | Yes | Yes | Yes | Yes | Yes | No |
| Jiang L, 2022 | Unclear | Yes | Yes | Yes | Yes | Yes | Yes | Yes | No |
| Li C, 2024 | Unclear | Yes | Yes | Yes | Yes | Yes | Yes | Yes | No |
| Liu X, 2024 | Yes | Yes | Yes | Yes | Yes | Yes | Unclear | Yes | No |
| Nam KW, 2017 | Yes | Yes | Yes | Yes | Yes | Yes | Yes | Yes | No |
| Qiao Y, 2025 | Yes | Yes | Yes | Yes | Yes | Yes | Yes | Yes | No |
| Wang Y, 2022 | Yes | Yes | Yes | Yes | Yes | Unclear | Yes | Yes | No |
| Wu Y, 2025 | Unclear | Yes | Yes | Yes | Yes | Yes | Unclear | Yes | Yes |
| Xiao CY, 2024 | Unclear | Yes | Yes | Yes | Yes | Yes | Yes | Yes | No |
| Yang J, 2025 | Yes | Yes | Yes | Yes | Yes | Yes | Yes | Yes | No |
| Zhao M, 2025 | Yes | Yes | Yes | Yes | Yes | Unclear | Yes | Yes | No |
| Nam KW, 2024 | Yes | Yes | Yes | Yes | Yes | Yes | Unclear | Yes | No |

**Supplemental Table 3. Sensitivity analyses for the association between NLR and CSVD after removing the corresponding studies.**

| **Study** | **Overall OR (95%CI)** | **I^2^** |
| --- | --- | --- |
| **presence of CSVD** |  |  |
| Cai J, 2024 | 1.25 (1.01, 1.56) | 62.2% |
| Jiang L, 2022 | 1.56 (1.07, 2.29) | 90.4% |
| Li C, 2024 | 1.36 (0.96, 1.92) | 90.7% |
| Wang Y, 2022 | 1.56 (1.07, 2.26) | 84.5% |
| **CSVD burden** |  |  |
| Chuang SY, 2023 | 1.46 (0.96, 2.21) | 67.1% |
| Jiang L, 2022 | 1.83 (1.41, 2.37) | 0.0% |
| Yang J, 2025 | 1.61 (1.03, 2.51) | 78.3% |
| Zhao M, 2025 | 1.40 (1.00, 1.97) | 63.0% |

NLR: neutrophil-to-lymphocyte ratio; CSVD: cerebral small vessel disease; OR: odds ratio; CI: confidence interval.

**Supplemental Table 4. Sensitivity analyses for the association between NLR and WMH severity and WMH volume, respectively, after removing the corresponding studies.**

| **Study** | **Effect size** | **I^2^** |
| --- | --- | --- |
| **WMH severity** | Overall OR (95%CI) |  |
| Cai J, 2024 | 1.22 (1.04, 1.42) | 50.7% |
| Chung D, 2020 | 1.37 (1.01, 1.85) | 90.3% |
| Jiang L, 2022 | 1.47 (1.12, 1.94) | 87.3% |
| Liu X, 2024 | 1.41 (1.01, 1.96) | 87.5% |
| Wu Y, 2025 | 1.34 (0.92, 1.93) | 90.3% |
| **WMH volume** | β (95%CI) |  |
| Fang Y, 2022 | 0.10 (0.00, 0.19) | 89.0% |
| Nam KW, 2017 | 0.06 (0.01, 0.11) | 80.2% |
| Qiao Y, 2025 | 0.11 (0.04, 0.18) | 71.7% |
| Xiao CY, 2024 | 0.09 (0.02, 0.17) | 89.8% |
| Nam KW, 2024 | 0.07 (0.01, 0.13) | 84.3% |

NLR: neutrophil-to-lymphocyte ratio; WMH: white matter hyperintensities; OR: odds ratio; CI: confidence interval.
